# In Union, Strength

**Published:** 1884-06

**Authors:** Henry N. Dodge

**Affiliations:** Morristown, N. J.


### IN UNION, STRENGTH.

BY HENRY N. DODGE, M. D., D. D. S., MORRISTOWN, N. J.

It might appear to the reader of current dental literature that the profession is torn into factions by a bitter feud between the advocates of the "New Departure" and the workers in gold alone. How little ground there is for such strife, and how often the two lines of practice may be advantageously united in a friendly alliance, is typified by the following unimportant incident of practice:

A superior second bicuspid, that recently came under my care, had decayed so extensively about a large gold filling that the pulp was dead and the mesial half of the tooth gone, the remainder of the crown being so much hollowed out that, excepting on the grinding surface, the walls of the cavity, or *cavern*, were the thinnest shell of enamel. The expediency of a large contour filling of cohesive gold extending below the gum, and in so frail a tooth, was doubtful, and the patient preferred to put off the use of a porcelain crown as the last resort. A gold screw, therefore, was firmly cemented into the root of the tooth with zinc phosphate, the free end of the screw extending nearly to the articulating surface of the crown, and the almost transparent walls of enamel received a thorough lining of the same plastic to increase their strength and render them opaque. That part of the cavity extending below the gum was then filled with Dr. Flagg's "sub-marine" amalgam, and the remainder of the cavity with his "usual" amalgam, packed carefully about the gold screw and finished with a "wafer" of the same material.

At a subsequent sitting, grooves and retaining points were cut into the amalgam and around the end of the gold screw, and all that part of the filling exposed to view was plated with cohesive gold, using both hand-pressure and the mallet in its manipulation, the contour being thus restored and the whole carefully finished. Every indication was met, and a frail tooth rendered once more serviceable and sightly.

